# COVID-19 and Diabetes: Persistent Cardiovascular and Renal Risks in the Post-Pandemic Landscape

**DOI:** 10.3390/life15050726

**Published:** 2025-04-30

**Authors:** Tzu-Shan Huang, Jo-Yen Chao, Ho-Hsiang Chang, Wei-Ren Lin, Wei-Hung Lin

**Affiliations:** 1 Department of Internal Medicine, College of Medicine, National Cheng Kung University Hospital, National Cheng Kung University, Tainan 704, Taiwan; n104491@mail.hosp.ncku.edu.tw (T.-S.H.); n053170@mail.hosp.ncku.edu.tw (J.-Y.C.); n049518@mail.hosp.ncku.edu.tw (W.-R.L.); 2 Division of Nephrology, Hualien Tzu Chi Hospital, Buddhist Tzu Chi Medical Foundation, Hualien 970, Taiwan; hl2300244@tzuchi.com.tw

**Keywords:** diabetes mellitus, COVID-19, cardiovascular disease, acute kidney injury, vaccination

## Abstract

The Coronavirus Disease 2019 (COVID-19) pandemic, caused by Severe Acute Respiratory Syndrome Coronavirus 2 (SARS-CoV-2), disproportionately affects individuals with diabetes mellitus (DM) by exacerbating cardiovascular and renal complications. This increased risk is mediated through immune dysfunction, chronic inflammation, hyperglycemia, dysregulation of renin-angiotensin system dysregulation, endothelial dysfunction, and hypercoagulability. Epidemiological studies indicate a two-fold increased risk of stroke and end-stage renal disease in SARS-CoV-2-infected individuals with diabetes, along with a 60% higher risk of cardiovascular disease. While antidiabetic therapies like sodium-glucose cotransporter-2 inhibitors and glucagon-like peptide-1 receptor agonists show potential protective effects, insulin use in hospitalized patients is linked to higher mortality. Vaccination is crucial in reducing severe COVID-19 outcomes and mitigating post-infection complications, including new-onset diabetes. While concerns exist regarding vaccine-associated nephropathy and thromboembolic events, these risks are thought to be minimal compared to the benefits. As COVID-19 shifts to an endemic phase, the long-term renal and cardiovascular outcomes in patients with DM remain uncertain, highlighting the urgent need for continued research and targeted management strategies.

## 1. Introduction

The Coronavirus Disease 2019 (COVID-19) pandemic, caused by the severe acute respiratory syndrome coronavirus 2 (SARS-CoV-2), emerged in late 2019 and rapidly evolved into a global health crisis. The virus spread rapidly across continents, overwhelming healthcare systems and causing millions of deaths worldwide [1]. Between 2020 and 2021, COVID-19 caused significant morbidity and mortality, particularly among vulnerable populations, including the elderly, immunocompromised individuals, and those with pre-existing metabolic disorders such as diabetes mellitus (DM) [2]. During this period, research focused primarily on acute disease management, including antiviral therapies, immunomodulatory treatments, and respiratory support strategies.

As of the post-pandemic era, the world has transitioned into a phase of coexistence with SARS-CoV-2, characterized by the widespread availability of vaccines, improved treatments, and endemic circulation of the virus [3]. Although acute infections have become less severe due to immunization efforts and antiviral advancements, emerging challenges related to long-term complications remain. Many patients continue to experience post-acute sequelae of SARS-CoV-2 infection, commonly referred to as “long COVID”, which includes persistent immune dysregulation, endothelial dysfunction, and organ impairment [4,5]. These complications are particularly concerning in individuals with underlying metabolic diseases such as DM, who are already at an elevated risk of cardiovascular and renal complications.

DM is a major risk factor for severe COVID-19 due to immune dysfunction, chronic inflammation, endothelial damage, and hyperglycemia [6]. Individuals with diabetes are more likely to experience severe illness, poorer prognosis, and higher mortality rates following SARS-CoV-2 infection [7,8]. DM is a key contributor to the cardiovascular–kidney–metabolic (CKM) syndrome, increasing the risk of cardiovascular disease (CVD) and serving as a predisposing factor for chronic kidney disease (CKD), which further heightens cardiovascular risk [9]. Although several studies have explored components of the relationship between COVID-19 and CKM-related conditions during the acute pandemic phase, the long-term interaction between the CKM syndrome and COVID-19—particularly in the post-pandemic era—remains insufficiently characterized [10,11,12].

Understanding the long-term impact of COVID-19 on renal and cardiovascular outcomes in diabetic patients is critical for optimizing future management strategies. In particular, exploring how different antidiabetic therapies, including sodium-glucose cotransporter-2 inhibitors (SGLT2is), glucagon-like peptide-1 receptor agonists (GLP-1RAs), metformin, dipeptidyl peptidase-4 inhibitors (DPP-4is), thiazolidinediones, α-glucosidase inhibitors, and insulin, as well as vaccination strategy, influence these outcomes will help refine treatment approaches in the post-pandemic setting.

## 2. COVID-19 and Cardiovascular Outcomes in Diabetes

COVID-19 significantly worsens cardiovascular prognosis, particularly in individuals with underlying conditions such as DM, CKD, obesity, and heart failure. These patients are more susceptible to severe cardiovascular outcomes, including acute kidney injury (AKI) and acute myocardial infarction (AMI) cardiac failure, stroke, accelerated atherosclerosis, venous and arterial thromboembolic disease [13,14]. COVID-19 is also linked to higher risk of arrhythmias, most commonly atrial fibrillation, as well as life-threatening ventricular tachycardia and ventricular fibrillation [15,16]. The mechanisms linking COVID-19 with cardiovascular complications are complex and multifactorial [13,14] (Table 1).

### 2.1. Pathophysiological Mechanisms

- **Renin-Angiotensin System (RAS) Dysregulation:** SARS-CoV-2 binds to ACE2 receptors, mainly located in proximal tubular cells, myocardium, and endothelial cells, which play a critical role in blood pressure regulation and cardiovascular homeostasis [13]. In patients with DM or CKD, baseline ACE2 expression is often downregulated, exacerbating RAS dysfunction and increasing the likelihood of hypertension and heart failure.
- **Endothelial Dysfunction and Inflammation:** Direct endothelial damage through ACE2 receptor binding leads to endothelial dysfunction, vascular inflammation, and microvascular thrombosis. Chronic hyperglycemia increases oxidative stress, further worsening cardiovascular complications [17]. Direct viral myocardial injury, systemic inflammation and hypoxia, may also cause arrhythmia, which is not necessarily secondary to cardiovascular complications such as AMI or heart failure [18].
- **Hypercoagulability and Pro-thrombotic State:** COVID-19 infection induces a systemic inflammatory response, with elevated levels of multiple cytokines (e.g., Tumor Necrosis Factor α [TNF-α], Interleukin-6), which contribute to acute thromboembolic events and long-term cardiovascular risk [19,20].

Persistent endothelial dysfunction, fibrosis, and increased cardiovascular risk have emerged as critical concerns, especially in patients with pre-existing comorbidities.

### 2.2. Epidemiological Evidence

Recent epidemiological studies have revealed that diabetes patients who contract COVID-19 face substantially heightened risks of adverse cardiovascular outcomes (Table 2):

- **Increased risk for Major Cardiovascular Event:** Findings from a self-controlled case series and matched cohort study in Sweden suggested an increased risk of AMI and ischemic stroke following COVID-19 [11]. Another nationwide study conducted in Denmark also found an increased risk of AMI and ischemic stroke in the acute phase of COVID-19 [21]. In the general diabetes population, compared to uninfected patients, those with COVID-19 had a two-fold increased risk of stroke and ESRD, along with a 60% higher adjusted risk of CVD [22,23]. Similar findings were observed in the Omicron-dominant cohort, where infected patients with diabetes had a higher likelihood of developing CVD, end-stage renal disease, stroke, and heart failure than their uninfected counterparts [23]. These findings underscore that the adverse cardiovascular impact of COVID-19 in diabetic individuals is both acute and sustained over time. Moreover, the interplay between the chronic inflammatory state inherent in diabetes and the acute inflammatory response induced by COVID-19 appears to amplify the risk of vascular complications. While the long-term cardiovascular effects of COVID-19 are still under active investigation, these findings emphasize the need for ongoing surveillance and targeted therapeutic strategies to mitigate the heightened cardiovascular risk in this vulnerable population.

**Table 2:** Cardiovascular and Renal Risks in Diabetes Patients Post-COVID-19.

Outcome	Infected DM Patients	Uninfected DM Patients
AMI Risk	↑ 2×	Baseline risk
Stroke Risk	↑ 2×	Baseline risk
Heart Failure	↑ 2.8×	Baseline risk
AKI	↑ 2.2×	Baseline risk
CKD Progression (eGFR decline)	↑ 6.15%	Baseline risk
ESRD Risk	↑ 2×	Baseline risk

↑ denotes an increased risk compared to baseline. **Key sources**: Studies from Korea, UK, Hong Kong, Canada (Refs. [22,23,24,25]).

- **Increased risk for arrhythmia:** A systematic review estimated arrhythmia prevalence in hospitalized COVID-19 patients at ~10.3%, with critically ill patients exhibiting a 12.1-fold higher risk [16]. According to the American Heart Association, these arrhythmias may result from direct cardiac injury, systemic inflammation, cytokine storm, and autonomic dysfunction [18]. They may occur independently or alongside complications like AMI or heart failure. Diabetes may further increase arrhythmia risk through metabolic disturbances, electrolyte imbalance, and inflammation—especially in the setting of diabetic ketoacidosis (DKA).
- **Increased risk for Peripheral Artery Disease (PAD):** PAD affects approximately 20% of patients with DM. Severe COVID-19 often manifests as a hyperinflammatory and prothrombotic condition, potentially impacting vascular health across multiple organ systems [26]. Emerging data suggest that adults with long COVID may have elevated peripheral arterial stiffness compared to controls [27]. Furthermore, a retrospective cohort study showed that individuals with prior COVID-19 infection exhibited a significantly increased incidence of PAD at both 3-month and 12-month follow-ups when compared to non-infected individuals [28]. While these findings raise concern, further research is warranted to determine the long-term PAD prognosis specifically in the diabetic population.
- **Intervention for Cardiovascular Event in COVID-19 patients:** In diabetic patients with COVID-19 experiencing acute cardiovascular events such as ST-elevation myocardial infarction, outcomes after conventional treatments (e.g., primary percutaneous coronary intervention) are notably worse than in non-diabetic counterparts [29]. These patients often exhibit higher thrombus burden, multivessel thrombosis, and increased risk of stent thrombosis, leading to poorer post-procedural TIMI flow [30,31]. The ISACS STEMI COVID-19 registry reported nearly double in-hospital and 30-day mortality in diabetic patients [29]. Additionally, hyperglycemia and delayed presentation during the pandemic have further exacerbated these poor outcomes by promoting inflammation, microvascular obstruction, and the no-reflow phenomenon [32,33]. Delayed presentation and prothrombotic milieu in the context of COVID-19 and diabetes likely contribute to these adverse outcomes. Further evaluation of late pandemic trends may be needed.

## 3. Diabetes and Renal Outcomes in COVID-19 Patients

DM significantly impacts renal function in the context of COVID-19. Studies have demonstrated that AKI is a common complication in hospitalized COVID-19 patients and is associated with worsened disease severity, prolonged hospitalization, and poor prognosis [24,34]. Patients with DM are particularly vulnerable to COVID-19-related kidney injury, with a higher incidence and greater severity of AKI compared to non-diabetic individuals [12].

### 3.1. Pathophysiological Mechanisms

- **Direct Kidney Injury:** SARS-CoV-2 can infect podocytes and proximal tubular cells via ACE2. The most common pathological finding is acute tubular injury followed with tubulointerstitial nephritis. Like other viral infections, glomerular lesion with protein leakage, podocyte injury is also noted. Among patients undergoing diagnostic kidney biopsy, the majority of patients present with collapsing focal segmental sclerosis, characterized by abrupt nephrotic range proteinuria; some also exhibit thrombotic microangiopathy. Mitochondrial dysfunction and other vascular lesions were also noted [13,35,36].
- **Systemic Inflammation and Coagulation Activation:** Cytokine storm-induced endothelial damage, coagulation activation, and disruption of the renin-angiotensin system exacerbate kidney injury and CKD progression [35,37].
- **Contributory Factors:** Hypoxia, hypotension, mechanical ventilation, and the use of nephrotoxic agents in critically ill COVID-19 patients may further contribute to kidney injury (Table 1).

A systematic review and meta-analysis indicated that patients with type 2 DM are at a two-fold increased risk of nephropathy and more severe AKI during COVID-19 infection, especially hospitalized patients, suggesting a heightened susceptibility to renal injury post-infection [24,38] (Table 2). Although there were some concerns regarding the potential impact of RAS inhibitors on kidney outcomes by modulating ACE2 expression, two large observational studies, such as the REPLACE COVID and BRACE CORONA trials, they found no clear association between the use of RAS inhibitors and the susceptibility or severity of SARS-CoV-2 infection or adverse kidney outcomes [39,40]. Consequently, RAS inhibitors can be safely continued in patients hospitalized with mild to moderate COVID-19.

As we transition into the post-pandemic era, treatment options for COVID-19 have matured, including drugs such as Paxlovid (Nirmatrelvir/Ritonavir), remdesivir, and tocilizumab, alongside rapid advancements in vaccination [41,42,43,44]. However, their protective effects on AKI and CKD, particularly in highly vulnerable populations such as those with DM, remain unclear. Notably, some reports have linked remdesivir and vaccination to AKI and nephropathy, but appear nonsignificant in meta-analysis [45,46].

### 3.2. Long-Term Outcome Considerations

- **eGFR decline in Long COVID:** Subgroup analysis of cohort about long-term effect of COVID-19 infection on kidney function among COVID-19 patients followed in post-COVID-19 recovery clinics in Canada showed that eGFR declined 6.15% after infection [25].
- **Post-COVID-19 Kidney Function Decline:** Large retrospective cohort studies reported that COVID-19-associated AKI was correlated with higher mortality and poorer long-term kidney function recovery, persistent elevations of baseline serum creatinine 125% up to one year, indicating sustained renal function impairment [38].
- **Long-Term Renal Outcomes:** A large retrospective cohort study found that among hospitalized patients, COVID-19-associated AKI was linked to a lower risk of long-term kidney function decline and all-cause mortality compared to influenza-related AKI and AKI from other critical illnesses [47]. In contrast, another cohort reported that hospitalized COVID-19 patients had a more rapid decline in kidney function than those hospitalized for pre-pandemic pneumonia [48]. The observed discrepancy appears to be primarily due to differing comparison groups. The former study compared hospitalized influenza with high mortality and other critical illness hospitalized patients during pandemic of COVID, critical illness-related AKI group included patients hospitalized for causes other than COVID-19, who may have been sicker and more prone to developing new chronic illnesses [47]. Notably, both studies observed post-discharge GFR decline, reinforcing the importance of long-term renal monitoring in this population [47,48].

These studies predominantly reflect data from the early pandemic period (2020–2021) and may not fully capture the evolving post-pandemic. Given the continued uncertainty regarding the long-term renal prognosis for patients with DM, it is crucial that COVID-19 survivors—particularly those who were hospitalized—receive closer monitoring of kidney function to enable early diagnosis and optimized management of CKD. Prospective longitudinal studies are needed to provide more definitive insights into renal outcomes in this high-risk population.

## 4. Antidiabetic Agent and COVID-19 Outcomes

The increased risk of COVID-19 in patients with DM is largely attributed to chronic hyperglycemia, pro-inflammatory states, and impaired immune responses, which predispose these individuals to both cardiovascular and renal complications. Maintaining optimal glycemic control is essential, and a variety of glucose-lowering therapies have been employed in clinical practice. These include SGLT2is, GLP-1RAs, metformin, DPP-4is, thiazolidinediones, α-glucosidase inhibitors, and insulin. In a series of recent meta-analyses, routine glucose-lowering therapies have been evaluated for their impact on COVID-19 outcomes, such as intensive care unit admission and mortality, in diabetic patients [1,49,50]. Studies indicate that metformin, GLP-1RAs, and SGLT-2is are associated with reduced mortality and fewer adverse events [1,51,52], while DPP-4is offer moderate benefits; conversely, insulin use is linked to a higher risk of poor outcomes. Despite the potential of SGLT2is to favorably modulate the cytokine storm seen in COVID-19, concerns over dehydration and euglycemic DKA persist [53], While liraglutide demonstrates anti-inflammatory effects, its theoretical risk of upregulating ACE2 warrants caution [54]. The higher risk of insulin may be attributed to the fact that insulin is often introduced in advanced diabetes stages, where patients have more comorbidities that independently contribute to poor outcomes. Additionally, studies recommend that patients already on oral antidiabetic agents should continue their current regimen rather than being switched to insulin during hospitalization for COVID-19, as maintaining pre-existing therapy may offer better glycemic control and reduce inflammation-associated complications [49]. The following sections will specifically discuss SGLT2is and GLP-1RAs, two glucose-lowering agents with established cardiovascular and renal benefits.

### 4.1. SGLT2is Usage in Patients with DM and COVID-19

SGLT2is, such as empagliflozin and dapagliflozin, block the SGLT2 protein responsible for about 90% of renal glucose reabsorption, thereby increasing urinary glucose excretion and reducing oxidative stress.

- **Neutral Impact on Clinical Outcomes:** In the DARE-19 trial, dapagliflozin did not significantly reduce new or worsening organ dysfunction, death, or improve clinical recovery in hospitalized COVID-19 patients with cardiometabolic risk factors [55]. Additionally, no significant difference was observed in a composite kidney endpoint (including AKI, initiation of kidney replacement therapy, or death) between patients with eGFR below or above 60 mL/min/1.73 m^2^, and the drug was well tolerated [56]. Similarly, the RECOVERY trial showed that empagliflozin did not significantly affect mortality, hospitalization duration, or progression to ventilation, while the ACTIV-4a trial revealed no significant improvement in days free of organ support or overall mortality with SGLT2s [57,58]. Across multiple randomized control trials, SGLT2s have shown a neutral effect on acute COVID-19 outcomes, though safety has been preserved.
- **Safety Profile and Caution for DKA:** Despite these neutral efficacy findings, SGLT2is were well tolerated and were not associated with a statistically increased risk of AKI or ketoacidosis, suggesting that routine discontinuation during hospitalization may not be necessary [55,58]. However, clinicians must remain aware of the possibility of euglycemic DKA, particularly in patients with COVID-19, where SARS-CoV-2–induced pancreatic toxicity may exacerbate ketogenesis, even in the context of normal or only mildly elevated blood glucose levels [53]. To enhance risk stratification and early detection, close monitoring of acid-base status and renal function is recommended during inpatient use of SGLT2is. In patients presenting with high anion gap metabolic acidosis, even in the absence of marked hyperglycemia—measurement of ketones is essential to rule out euglycemic DKA. Upon diagnosis, prompt discontinuation of the SGLT2is and initiation of insulin therapy with appropriate supportive care are critical. Additional risk factors such as prolonged fasting or dehydration should be carefully assessed to guide individualized management decisions in the context of COVID-19-related illness [53].
- **Long-Term Outcome Considerations:** Although SGLT2is have demonstrated benefits in improving glycemic control and reducing oxidative stress, their potential impact on COVID-19 survivors remains to be fully defined [59,60]. Therefore, long-term evidence for these benefits in the context of COVID-19 is still lacking and requires further evaluation [59].

### 4.2. GLP-1RAs Usage in Patients with DM and COVID-19

GLP-1RAs, including long-acting agents such as liraglutide, dulaglutide, and semaglutide, as well as short-acting agents like exenatide and lixisenatide, are incretin mimetics that enhance glucose-dependent insulin secretion, suppress glucagon release, and delay gastric emptying.

- **Potential Benefit due to Antiinflammation and Other Mechanisms:** Beyond their glycemic effects, GLP-1RAs exhibit anti-inflammatory and antioxidant properties by inhibiting NF-κB-dependent cytokine release, reducing pro-inflammatory cytokines such as interleukin-1β and TNF-α. These effects suggest a potential role in mitigating systemic inflammation and oxidative stress in COVID-19 patients [61]. Additionally, GLP-1RAs may upregulate ACE2 expression, counteracting its SARS-CoV-2-mediated downregulation and potentially reducing lung injury.
- **Reduced Mortality including Cardiovascular Deaths:** Observational studies have provided mixed findings regarding GLP-1RAs in COVID-19. Meta-analysis of observational studies indicated significant mortality benefits with preadmission GLP-1RAs use, warranting further investigation through randomized controlled trials [1,62]. Secondary analysis from the SELECT trial showed that semaglutide (2.4 mg) reduced all-cause mortality, including cardiovascular and non-cardiovascular deaths, in treated patients [63]. While semaglutide did not lower COVID-19 incidence, it was associated with fewer COVID-19-related serious adverse events and deaths among infected individuals [63]. There is also evidence that GLP-1RA may have therapeutic effect on COVID-19 induced pulmonary artery hypertension, through anti-inflammatory, antioxidant, and vasoregulatory effects [64]. GLP-1RAs suppress pro-inflammatory cytokines such as IL-6 and TNF-α, reducing pulmonary inflammation and cytokine storm severity [65,66]. They also decrease oxidative stress and mitochondrial dysfunction by downregulating Drp1/Nox and autophagy-related pathways in pulmonary arterial smooth muscle cells, limiting vascular remodeling [67]. Furthermore, they upregulate ACE2 expression, restoring renin–angiotensin system balance and alleviating right ventricular strain [68].
- **Long Term Benefits and Therapeutic Role of GLP-1RAs:** Despite these promising findings, routine use of GLP-1RAs specifically for COVID-19 management is not yet justified, pending further randomized control trials. Evidence of long-term benefits still needs further investigations. However, their established benefits of cardiovascular and renal protection support continued use in patients with DM, with potential added advantages in mitigating COVID-19 complications [69,70].

## 5. COVID-19 Vaccination in Diabetes Patients

The Centers for Disease Control and Prevention recommends a three-dose primary series of mRNA vaccines (Moderna, Pfizer-BioNTech) or Novavax, with an additional dose for the 2024–2025 variant. Individuals with DM should receive the full vaccine series for protection against severe COVID-19 outcomes [71]. However, vaccine hesitancy remains a concern, particularly among patients with pre-existing kidney disease, underscoring the need for targeted public health interventions.

### Vaccine Benefits vs. Risk

- **Protective Effects:** Vaccination reduces the risk of severe COVID-19, hospitalization, and mortality in patients with DM.
- **Potential Risks:** There are reports of vaccine-associated nephropathy, myocarditis, and thromboembolic events; however, systematic reviews suggest these risks are minimal compared to the benefits [72,73,74,75].
- **New-Onset Diabetes Post-Infection Risk reduced after Vaccination:** Emerging evidence indicates that the risk of new-onset diabetes is significantly elevated following SARS-CoV-2 infection, particularly in unvaccinated individuals. A recent cohort study by Kwan et al. found that COVID-19 vaccination was associated with a lower risk of developing diabetes post-infection, reinforcing its role in mitigating metabolic complications [76]. SARS-CoV-2 has been linked to pancreatic beta-cell dysfunction, systemic inflammation, and insulin resistance, all of which contribute to the development of new-onset diabetes. This includes both hyperglycemia in previously healthy individuals and worsening glycemic control in those with pre-existing diabetes, with complications such as diabetic ketoacidosis and hyperosmolar hyperglycemic syndrome reported. Meta-analyses and cohort studies have consistently shown increased rates of hyperglycemia in both diabetic and non-diabetic individuals following COVID-19 [77,78,79,80]. Vaccination thus emerges as a critical intervention in reducing the incidence of new-onset diabetes and its complications following COVID-19 [8,76].

## 6. Future Directions

DM substantially increases the risk of adverse outcomes following COVID-19 infection. During the early pandemic, hospitalized patients with DM exhibited significantly elevated rates of hospital mortality, stroke, cardiovascular events, and acute kidney injury [10]. Future research should focus on long-term cardiovascular and renal risks in diabetic patients following COVID-19, especially in the later phases of the pandemic. In particular, it is important to assess the potential protective roles of therapies such as SGLT2 inhibitors and GLP-1 receptor agonists. Furthermore, there is a critical need to explore the mechanisms underlying viral persistence, immune dysregulation, and recurrent infections to optimize post-infection management strategies. A comprehensive precision medicine approach could enhance intervention tailoring in this high-risk population. For example, genetic risk profiling may be utilized to identify individuals predisposed to severe organ complications post-COVID-19, while pharmacogenomic studies could elucidate how genetic variations affect responses to treatments, including the efficacy and adverse reaction profiles of SGLT2is and GLP-1RAs. In addition, digital monitoring strategies, including continuous glucose monitors and wearable devices for heart rate, oxygen saturation, and rhythm tracking, may facilitate the early detection and proactive management of post-COVID complications. Collectively, these targeted approaches, combined with systematic analyses of long-term cardiovascular and renal outcomes from real-world vaccination data, will be essential for developing tailored therapeutic strategies aimed at improving clinical outcomes for diabetic patients affected by COVID-19.

## 7. Conclusions

DM amplifies COVID-19-associated cardiovascular and renal complications through RAS dysregulation, endothelial dysfunction, and systemic inflammation, further contributing to CKM syndrome. While short-term outcomes, including AKI and cardiovascular events, are poor, long-term renal prognosis remains debated. As treatments and vaccines evolve, further research is needed to assess their impact on patients with DM. Despite concerns about potential side effects, vaccines significantly reduce severe illness, hospitalization, and mortality, and may lower post-infection diabetes and cardiovascular risks. Ongoing monitoring and targeted management strategies are crucial for improving outcomes in this high-risk population in the post-pandemic period.

## Figures and Tables

**Table 1:** Mechanisms Linking COVID-19, Diabetes, and CKM Syndrome.

Pathophysiology	Effects in DM Patients	Clinical Implications
RAS Dysregulation	↑ Hypertension, ↑ Inflammation	Higher cardiovascular risk
Endothelial Dysfunction	Microvascular thrombosis, ↑ Oxidative stress	Increased AMI and stroke risk
Cytokine Storm and Chronic Inflammation	↑ IL-6, TNF-α, persistent immune activation	Arrhythmia, Organ fibrosis, CKD progression
Hypercoagulability and Pro-thrombotic State	↑ D-dimer, platelet aggregation	Risk of thromboembolic events
Direct Kidney Injury (via ACE2 Receptors)	Acute tubular necrosis, podocyte injury	Higher risk of AKI and ESRD

↑ indicates an increase or elevation. Abbreviation: DM, Diabetes mellitus; RAS, Renin-Angiotensin System; AMI, Acute Myocardial Infarction; IL-6, Interleukin-6; TNF-α, Tumor Necrosis Factor α; CKD, Chronic Kidney Disease; AKI, Acute Kidney Injury; ESRD, End-stage renal disease.

## Data Availability

No new data were created or analyzed in this study. Data sharing is not applicable to this article.

## Abbreviations

The following abbreviations are used in this manuscript:

COVID-19	Coronavirus Disease 2019
SARS-CoV-2	Severe Acute Respiratory Syndrome Coronavirus 2
DM	Diabetes Mellitus
eGFR	Estimated Glomerular Filtration Rate
RAS	Renin-Angiotensin System
CKM	Cardiovascular–Kidney–Metabolic Syndrome
CVD	Cardiovascular Disease
CKD	Chronic Kidney Disease
ESRD	End-Stage Renal Disease
AKI	Acute Kidney Injury
AMI	Acute Myocardial Infarction
DKA	Diabetic Ketoacidosis
TNF-α	Tumor Necrosis Factor Alpha
PAD	Peripheral Arterial Disease
SGLT2is	Sodium-glucose cotransporter-2 inhibitors
GLP-1RAs	Glucagon-like peptide-1 receptor agonists
DPP-4is	Dipeptidyl peptidase-4 inhibitors
